# Gene expression profiling in circulating endothelial cells from systemic sclerosis patients shows an altered control of apoptosis and angiogenesis that is modified by iloprost infusion

**DOI:** 10.1186/ar3069

**Published:** 2010-07-07

**Authors:** Elisa Tinazzi, Marzia Dolcino, Antonio Puccetti, Antonella Rigo, Ruggero Beri, Maria Teresa Valenti, Roberto Corrocher, Claudio Lunardi

**Affiliations:** 1 Section of Internal Medicine B, Department of Medicine, University of Verona, P.le LA Scuro, 10, 37134, Verona, Italy; 2 Immunology Unit, Institute G. Gaslini, Largo G. Gaslini, 16147, Genova, Italy; 3 Section of Histology, Department of Experimental Medicine, University of Genova, Via Marsano 10, 16132, Genova, Italy; 4 Section of Hematology, Department of Medicine, University of Verona, P.le LA Scuro, 10, 37134, Verona, Italy; 5 Section of Internal Medicine D, Department of Medicine, University of Verona, P.le LA Scuro, 10, 37134, Verona, Italy

## Abstract

**Introduction:**

Circulating endothelial cells are increased in patients affected by systemic sclerosis (SSc) and their number strongly correlates with vascular damage. The effects of iloprost in systemic sclerosis are only partially known. We aimed at studying the gene expression profile of circulating endothelial cells and the effects of iloprost infusion and gene expression in patients with systemic sclerosis.

**Methods:**

We enrolled 50 patients affected by systemic sclerosis, 37 patients without and 13 patients with digital ulcers. Blood samples were collected from all patients before and 72 hours after either a single day or five days eight hours iloprost infusion. Blood samples were also collected from 50 sex- and age-matched healthy controls. Circulating endothelial cells and endothelial progenitors cells were detected in the peripheral blood of patients with systemic sclerosis by flow cytometry with a four-colour panel of antibodies. Statistical analysis was performed with the SPSS 16 statistical package.Circulating endothelial cells were then isolated from peripheral blood by immunomagnetic CD45 negative selection for the gene array study.

**Results:**

The number of both circulating endothelial cells and progenitors was significantly higher in patients affected by systemic sclerosis than in controls and among patients in those with digital ulcers than in patients without them. Circulating endothelial cells and progenitors number increased after iloprost infusion. Gene array analysis of endothelial cells showed a different transcriptional profile in patients compared to controls. Indeed, patients displayed an altered expression of genes involved in the control of apoptosis and angiogenesis. Iloprost infusion had a profound impact on endothelial cells gene expression since the treatment was able to modulate a very high number of transcripts.

**Conclusions:**

We report here that circulating endothelial cells in patients with systemic sclerosis show an altered expression of genes involved in the control of apoptosis and angiogenesis. Moreover we describe that iloprost infusion has a strong effect on endothelial cells and progenitors since it is able to modulate both their number and their gene expression profile.

## Introduction

Systemic sclerosis (SSc) is a rare systemic autoimmune disease characterized by a preminent vascular endothelial dysfunction, by immunological abnormalities, and by excessive extracellular matrix accumulation leading to fibrosis of the skin and internal organs [1].

Endothelial cell (EC) damage defines a crucial step during the pathogenesis of vascular disorders since its injury leads to the loss of the anti-thrombotic properties of the vessels wall and rapidly enhances the number of damaged circulating endothelial cells (CECs). CECs are likely to represent those cells shed from vascular luminal endothelium as a result of insults in disease states [2]. They correlate with physiological markers of endothelial damage/dysfunction and they have been identified as a marker of vascular damage in a variety of disorders, including malignancy, cardiovascular diseases and autoimmune disorders such as systemic sclerosis (SSc) and vasculitides [3-9]. In healthy subjects, CECs are rarely detectable and probably represent the effect of natural endothelial cells turnover [10]. Therefore, complete regeneration of injured endothelium is of particular importance and may occur by migration and proliferation of surrounding mature endothelial cells.

CECs are terminally differentiated cells with a low proliferative potential and their capacity to substitute damaged endothelial cells and to create new vessels is relative limited [11]. Moreover accumulating evidence indicates that bone marrow-derived progenitor cells have the potential to differentiate into mature CECs and they have been termed endothelial progenitor cells (EPCs) [12-15]. As a consequence, EPCs can give an effective contribution to endothelization and neo-vascularization as shown by different studies in animal models and humans [10,16-18].

Iloprost, a chemically stable prostacyclin analog [19], has been shown to induce long-term clinical improvement in various vascular conditions, including ischemic ulcers and pulmonary hypertension primary or secondary to SSc [20]. Iloprost infusion increases arteriolar distension and blood flow as a result of a vasodilating effect. The drug inhibits platelet activation and aggregation, and leukocyte activity [21]. Iloprost therapy has also a protective and reparatory effect by influencing EPCs [22]. The pharmacological effect on ECs modulates the adhesion molecules (E-selectin, ICAM-1, VCAM-1) expression and growth factors release, particularly VEGF and CTGF [23,24].

The biological activity is mediated by a specific interaction with the I prostanoid (IP) membrane receptor [25], the same receptor as prostaglandin I_2_. Iloprost is a potent IP receptor agonist that activates adenylate cyclase, resulting in an acute increase in intracellular cyclic AMP. Such an increase in cAMP has profound effects on cellular function in platelets, endothelial cells, smooth muscle cells, fibroblasts, and in a number of different cell types involved in both innate and acquired immunity [23,24,26,27]. We reasoned that such a strong impact on the function of different cell types and particularly of endothelial cells is the result of the modulation of several genes, an aspect that has never been looked at, *in vivo*.

We therefore aimed to evaluate the role played by iloprost infusion on circulant endothelial cell number and to clarify the molecular effects of the treatment in patients with SSc by studying CECs gene expression profiling before and after the treatment. Moreover, since digital ulcers are the key clinical manifestation of severe vascular damage, we considered a group of patients with skin ulcers separately, in order to evaluate whether in this subset of patients both the numbers and the gene expression of CECs is different from patients with a less severe vascular involvement.

## Materials and methods

### Patients and controls

We enrolled 50 patients affected by SSc: 37 without skin ulcers and 13 with digital ulcers; 18 patients were affected by the diffuse cutaneous form and 32 by the limited cutaneous form of the disease. Fifty age- and sex-matched healthy donors were enrolled as controls.

Blood samples collected in EDTA using a Vacutainer system (Becton Dickinson, NJ, USA) were drawn from patients before, and 72 hours after a single day or five days of being infused with iloprost for eight hours. In both cases the first 7 ml of blood was discarded and blood was processed within three hours after collection.

The study was approved by the local ethics committee (Comitato Etico per la Sperimentazione, Azienda Ospedaliera Universitaria di Verona) and informed written consent was obtained from all the participants to the study.

### Detection of circulating endothelial cells and progenitors by flow-cytometry

CECs and EPCs were directly detected in whole peripheral blood in EDTA by *lyse-no-wash *method. Two hundred μL of each sample were incubated with a mixture of monoclonal antibodies for 20 minutes at room temperature after a 10-minute preincubation with a *blocking *serum. Fluorescein isothiocyanate (FITC)-conjugated anti-CD45, R-Phycoerythrin (PE)-conjugated anti-CD146, -CD31, -CD133 and -CD34 or isotype-matched control (IgG_1_), allophyco-cyanine (APC)-conjugated anti-CD3, -CD16, -CD19 and -CD33 were used. 7-Amino-actinomycin D (7-AAD) was added for dead cells exclusion. Samples were also stained with anti-CD45 FITC, anti-CD146, -CD31, -CD133, -CD34 PE, anti-CD106 or anti-VEGFR2 APC and peridin-chlorophill-protein (PerCP)-conjugated anti-CD3, -CD16, -CD19 and -CD33.

All reagents were purchased from Becton Dickinson (San Jose, CA, USA), except for anti-CD16 (Caltag, Burlingame, CA, USA), anti-CD106 (Biolegend, San Diego, CA, USA) and anti-VEGFR2-APC (R & D Systems, Minneapolis, MN, USA)

After labeling, red blood cells were lysed by incubation with 2 ml of ammonium chloride solution. The samples were analysed on a FACS Calibur cytometer (Becton Dickinson). The sensitivity of fluorescence detectors was set and monitored using Calibrite Beads (Becton Dickinson) according to the manufacturer's recommendations; 500.000 cells per sample were acquired in live gating. FlowJo 8.8.2 software (Tree Star, Ashland, OR, USA) was used to analyze data. A sequential Boolean gating strategy [28], designed to remove dead cells, platelet aggregates and debris, and to exclude CD45 + and CD3 +/CD19 +/CD16 +/CD33 + hematopoietic cells (dump channel), was used to accurately enumerate total CECs and EPCs [29]. The absolute number of CECs and EPCs was established in double platform, combining the flow-cytometrically assessed per cent cells and the white blood cells (WBC) count assessed using a haematology cell analyser [30].

### Isolation of CECs and EPCs from peripheral blood

Twenty ml of blood obtained from all patients were added to 40 ml of phosphate buffered saline (PBS) solution. Mononuclear cells were isolated by density gradient centrifugation using Ficoll-Paque, washed twice with PBS and suspended in 80 μl of degassed separation buffer (PBS pH 7.2, 0.5% BSA, 2 mM EDTA) per 10^7 ^cells. Cells were incubated with 20 μl of anti-CD45 coated immunomagnetics micro-beads (Miltenyi Biotech, Auburn, CA, USA) for 15 minutes at 4°C with gentle rotation. Bead-bound cells were then separated from unbound cells by a magnetic sorting on LD columns (Miltenyi). CECs and EPCs were found in the fraction of unbound cells (CD45 low/negative). An aliquote of each fraction was analyzed by FACS using anti-CD45 FITC, anti-CD146/CD31/CD34/CD133 PE and 7-AAD to confirm the endothelial origin and quantify the possible lymphocyte contamination.

### RNA extraction

We obtained CECs and EPCs from peripheral blood of 13 patients affected by SSc with digital ulcers and 37 patients without any skin ulcer before, and 72 hours after, iloprost infusion. Cells within each patient's group were counted and pooled together for RNA extraction. Each patient contributed to the pool with the same number of CECs. Control RNA was extracted from circulating endothelial cells (CECs + EPCs) obtained from 50 healthy donors.

### Gene array analysis

Cell pellets of CECs and EPCs obtained from SSc patients, with and without digital ulcers, before and 72 hours after iloprost infusion both after one and five days of therapy (test samples) were used for gene array experiments. CECs and progenitors purified from healthy donors were used as control samples.

Isolation of total RNA, preparation of cRNA, hybridization, and scanning of probe arrays were performed according to the protocols of the manufacturer (Affymetrix, Santa Clara, CA, USA) by Cogentech (Consortium for Genomic Technologies c/o IFOM-IEO Campus, Milano, Italy). To ensure that a sufficient amount of cDNA was available, the RNA extracted from CECs was subjected to a two-cycle cDNA synthesis according to Affymetrix protocol. Biotinylated target cRNA was hybridized to the Human Genome U133A 2.0 GeneChip (Affymetrix). The Human Genome U133A GeneChip is a single array representing 14,500 well-characterized human genes and includes more than 22,000 probe sets and 500,000 distinct oligonucleotide features.

The different gene expression patterns were analyzed using Array Assist version 5.0 (Stratagene, La Jolla, CA, USA), which calculates background-adjusted, normalized, and log-transformed intensity values applying the PLIER algorithm [31-33].

The PLIER method uses quartile normalization and runs an optimization procedure which determines the best set of weights on the perfect match (PM) and mismatch (MM) for each probe pair. Finally, the normalized, background-corrected data were transformed to the log2 scale. A signal log2 ratio of 1.0 indicates an increase of the transcript level by two-fold change (2 F.C.) and -1.0 indicates a decrease by two-fold (-2 F.C.). A signal log2 ratio of zero would indicate no change.

Genes were selected for final consideration when their expression (F.C.) was at least two-fold different in the test sample versus the control sample. Experiments were performed in duplicates [34].

Selected genes were submitted to a functional classification according to the Gene Ontology (GO) annotations [35]. To find the GO terms overrepresented in our dataset, a GO enrichment was calculated with Array Assist that operates a statistical computation using a hypergeometric distribution [36].

### Real time RT-PCR

Total RNA was extracted from endothelial cells using TRIzol reagent (Invitrogen, Carlsbad, CA, USA), following manufacturer's instructions. First-strand cDNA was generated using the SuperScript III First-Strand Synthesis System for RT-PCR Kit (Invitrogen), with random hexamers, according to the manufacturer's protocol. RT product was aliquoted in equal volumes and stored at -20°C.

PCR was performed in a total volume of 25 μl containing 1× Taqman Universal PCR Master mix, no AmpErase UNG and 2.5 μl of cDNA; pre-designed, Gene-specific primers and probe sets for each gene (BCL2 Hs99999018-m1) (ICAM1 Hs00164932-m1) (VEGFA Hs00900055-m1) were obtained from Assay-on-Demande Gene Expression Products (Applied Biosystems).Real Time PCR reactions were carried out in a two-tube system and in singleplex. The Real Time amplifications included 10 minutes at 95°C (AmpliTaq Gold activation), followed by 40 cycles at 95°C for 15 seconds and at 60°C for one minute. Thermocycling and signal detection were performed with ABI Prism 7300 Sequence Detector (Applied Biosystems). Signals were detected according to the manufacturer's instructions. This technique allows the identification of the cycling point where PCR product is detectable by means of fluorescence emission (Threshold cycle or Ct value). As previously reported, the Ct value correlates to the starting quantity of target mRNA [37]. Relative expression levels were calculated for each sample after normalization against the housekeeping gene GAPDH, using the ΔΔCt method for comparing relative fold expression differences [38]. The data are expressed as mRNA fold.

Ct values for each reaction were determined using TaqMan SDS analysis software. For each amount of RNA tested triplicate Ct values were averaged. Because Ct values vary linearly with the logarithm of the amount of RNA, this average represents a geometric mean.

### Statistical analysis

Calculations were performed with the SPSS 16 statistical package. Comparison of CECs and EPCs levels between healthy controls and patients affected by SSc with and without ulcers were performed by T-test and Pearson test. Correlations between CECs and EPCs number before and after iloprost infusion were assessed with a non parametric test (Wilcoxon test).

Comparison of gene expression by Real Time RT-PCR was carried out by T-test.

## Results

### CECs and EPCs in patients with SSc

CECs and EPCs are extremely rare in the peripheral blood of healthy people, representing somewhere between 0.01% and 0.0001% of mononuclear cells [11,29]. Flow-cytometry offers the advantage of a rapid and accessible technique [29,30], with the availability of multiple markers as well as the possibility of distinguishing CECs and EPCs using a small blood volume.

Key elements for accurate detection and enumeration of rare events in flow cytometry are the number of events acquired and the signal to noise ratio. Collection of a large number of events is mandatory to identify an adequate number of a rare event population; therefore, we stored 500,000 cells per sample in live gating. To minimize noise, we reduced non-specific binding by preincubating cells with blocking serum and doublets acquisition by an adequate flow rate. Dead cells can be a major source of non-specific staining by monoclonal antibodies. A real-time viability stain (7-AAD) was used to identify dead cells and to exclude them from analysis. We also established a dump channel (CD3, CD16, CD19, CD33) to exclude cells not of interest for the analysis. Indeed, the interest of the method reported here lies in the high intra-assay reproducibility and the high precision in the detection of both CECs and EPCs due to the gating strategy and to the presence of a dump channel [39,40].

Finally, since no markers are entirely specific for endothelial cells, we used a multicolour approach and to maximize the signal we used the best fluorochrome (PE) for the most critical detection. CD146 and CD31 are useful as endothelial cell markers and were used in combination, since both these markers are individually expressed by other cell types, such as activated T-lymphocytes, pericytes, bone marrow fibroblasts, nerve fibers and leukocytes subsets and platelet/leukocytes aggregates respectively [41]. CD34, CD133 and VEGFR2 were used to more precisely identify EPCs.

CECs were defined as CD45 negative, CD146/CD31/CD34 positive and CD133 negative. EPCs are greater than CECs and are CD146/CD31 negative, CD34/CD133 positive, CD45 low positive and VEGFR2 positive [29].

Evaluation of CECs and EPCs by flow-cytometry showed that the number of CECs and EPCs were significantly higher in SSc patients than in controls and that among patients, CECs were higher in patients with cutaneous ulcers than in those without ulcers. The difference in CECs and EPCs numbers was statistically significant when SSc patients were compared to healthy controls (Table 1); such difference was significant only for CECs in SSc patients with skin ulcers versus patients without ulcers (Table 2). Patients with the cutaneous limited form of the disease showed no statistical difference in CEC and EPC numbers compared to the patients with the diffuse cutaneous form, even if FACS analysis showed a trend towards an increased number of CECs and EPCs in patients with the diffuse cutaneous form (data not shown).

**Table 1:** Comparison of CECs and EPCs number between patients affected by SSc and healthy controls

	SSc patients (50)	Healthy controls (50)	*P *value
CECs/mmc	689 ± 464	22 ± 17	< 0,0001
EPCs/mms	146 ± 92	1 ± 1	< 0,0001

CECs, circulating endothelial cells; EPCs, endothelial progenitor cells; SSc, progressive systemic sclerosis.

**Table 2:** Comparison of CECs and EPCs number between patients with and without skin ulcers

	Skin ulcers - SSc patients (33)	Skin ulcers + SSc patients (17)	*P *value
CECs/mmc	600 ± 401	968 ± 553	0.05
EPCs/mms	142 ± 93	158 ± 92	0.597

CECs, circulating endothelial cells; EPCs, endothelial progenitor cells; SSc, progressive systemic sclerosis.

**Figure 1:**
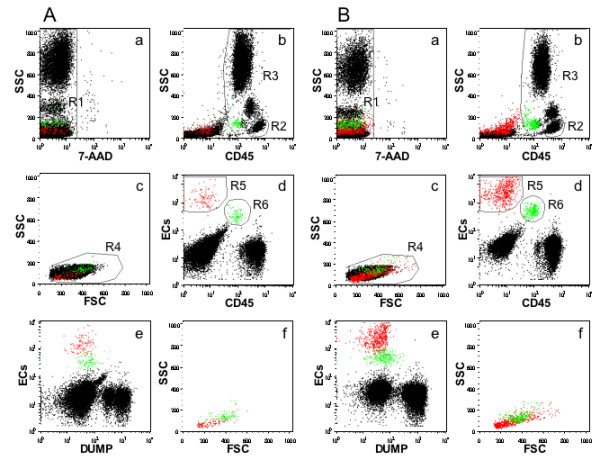
**FACS analysis of ECs detected in a patient affected by Systemic Sclerosis**. Panel A: Before iloprost infusion; Panel B: After iloprost infusion. Sequential four-color gating strategy. In cytogram (a) which displays all events, a rectangular region (R1) is drawn to exclude dead cells from analysis (7-AAD positive-cells). In cytogram (b), a polygonal region (R2) is drawn to define lymphocytes on the basis of the morphological parameter Side Scatter (SSC) and of CD45 expression. An additional region (R3), which includes all CD45 positive events, is depicted to derive CECs and EPCs enumeration. In cytogram **(c)**, R4 is defined as FSC (Forward Scatter)/SSC gate on lymphocytes set on FSC left-hand border and include intermediate region between lymphocytes and monocytes. In cytogram **(d) **are included all events which meet morphological criteria of R4. R5 and R6 include respectively CECs and EPCs which are shown negative for dump channel markers (CD3/CD16/CD19/CD33) in cytogram **(e)**. CECs and EPCs show a different staining with CD146/CD31/CD133/CD34 (ECs). Cytogram *(f) *shows the morphological characteristics of cells in R5 and R6 (CECs and EPCs respectively).

We observed an increased number of CECs and EPCs in patients after iloprost infusion (Figure 1A, B) with a statistically significant difference in CECs count only when the comparison was performed before and 72 hours after the five days' iloprost infusion (*P-*value 0.004) while EPCs count showed a statistically significant difference both after one and five days of therapy (Table 3).

**Table 3:** Number of CECs and EPCs before and after iloprost infusion

	CECs/mmc	EPCs/mmc
Data before iloprost infusion	661 ± 404	152 ± 93
Data 72 h after one day iloprost therapy	745 ± 453	186 ± 104^¶^
Data 72 h after five days iloprost therapy	775 ± 382	206 ± 139^¥^

* *P*-value 0.368 vs cells number before iloprost infusion

# *P*-value 0.004

¶ *P*-value 0.015

^¥ ^*P*- value 0.014

CECs, circulating endothelial cells; EPCs, endothelial progenitor cells.

Taken together, these data indicate that the CECs and EPCs count is significantly higher in patients compared to healthy controls and that iloprost infusion induces a significant enrichment in both cell populations.

### Gene array analysis of endothelial cells

We decided to use a gene array approach to analyse the transcriptional profiles of CECs in SSc patients. Since the purification procedure allows the recovery of a very limited amount of cells, our samples were prepared by mixing both EPCs and CECs, therefore, from now on and for this set of experiments, the term CECs will refer to the cell population that includes the two cell subtypes.

CECs were isolated from 37 patients without ulcers and from 13 patients with ulcers. CECs obtained from each group of subjects were then pooled for RNA extraction. Each patient contributed to the pooled sample with the same number of cells. CECs were also isolated from the blood of 50 healthy donors.

We compared the gene expression patterns of CECs obtained from SSc patients either in presence or in absence of digital ulcers with those obtained from normal healthy donors. As described in the Methods section only those genes modulated more than two-fold compared to the control sample (normal healthy donors) were considered in our analysis.

All the results of the gene array analysis have been deposited in the public repository ArrayExpress (accession number: [E-MEXP-2769]).

In CECs from patients with ulcers 6,544 genes were modulated when compared to the healthy counterpart, in particular 5,260 transcripts were down-regulated and 1,284 genes were up-regulated (Additional files 1, 2, 3).

A profound difference in gene expression was also observed in CECs obtained from patients without ulcers with 6,672 modulated genes (5,425 down-regulated genes and 1,247 up-regulated genes) (Additional files 4, 5).

These data showed that the transcriptional profiles of CECs in SSc were profoundly different from the transcriptional profiles of CECs of healthy donors, indicating that the two populations were quite heterogeneous at least at transcriptional level.

Among the genes differently expressed in these two populations, the number of down-regulated genes was significantly higher when compared to the number of the up-regulated ones.

CECs were also obtained from the same patients 72 hours after treatment with iloprost and the gene expression profiles of these cells were compared to the ones of CECs obtained from the same patients before treatment.

The treatment resulted in differential expression of 2,133 genes (1,080 up-regulated and 1,053 down-regulated) in patients with digital ulcers (Additional files 6, 7). A higher number of genes (6,643) was modulated by the iloprost infusion in patients without digital ulcers: the up-regulated were 5,081, while the down-regulated ones were 1,562 (Additional files 8, 9).

The results so far obtained showed that iloprost treatment had a strong impact on the transcriptional activity of CECs derived from SSc patients with and without digital ulcers.

Given the high number of modulated genes, we next decided to focus our attention on the effect of the treatment on the genes differently expressed in patients affected by SSc versus healthy donors. We therefore selected within the 6,544 transcripts differently expressed in patients with digital ulcers only those genes which were also modulated after iloprost treatment in the same patients. This subset of genes included 1,211 transcripts.

We then performed a Gene Ontology (GO) analysis to cluster genes into functional classes according to GO biological processes and molecular functions and selected the functional classes overrepresented among the differentially expressed genes (GO term enrichment). The modulated genes belong to several functional classes including: positive regulation of anti-apoptosis, response to stress, response to wounding and wound healing, Wnt receptor activity, receptor complex, membrane, chemotaxis, DNA-dependent DNA replication, prostaglandin-reductase activity, G0 to G1 phase transition, platelet-derived growth factor beta-receptor activity, actin cytoskeleton organization and biogenesis, innate immune response. Representative examples of such genes within the above mentioned functional classes are presented in a compiled form in Table 4 which includes Gene Bank accession numbers and F.C. of expression of the genes.

**Table 4:** Functional classification of genes modulated by iloprost in SSc patients with digital ulcers

Probe set ID	Gene Title	Gene symbol	F.C. SSc ulcers/healthy	F.C. SSc ulcers post-treatment/SSc ulcers pre-treatment	Representative Public ID
**Positive regulation of anti-apoptosis**					
210621_s_at	RAS p21 protein activator (GTPase activating protein) 1	RASA1	8.72 down	4.28 up	M23612
214917_at	protein kinase, AMP-activated, alpha 1 catalytic subunit	PRKAA1	6.49 down	6.60 up	AK024252
201849_at	BCL2/adenovirus E1B 19 kDa interacting protein 3	BNIP3	69.04 down	11.78 up	NM_004052
**Response to stress**					
200985_s_at	CD59 molecule, complement regulatory protein	CD59	18.76 down	2.72 up	NM_000611
202906_s_at	nibrin	NBN	12.38 down	4.28 up	AF049895
206040_s_at	mitogen-activated protein kinase 11	MAPK11	17.27 up	10.62 down	NM_002751
209305_s_at	growth arrest and DNA-damage-inducible. beta	GADD45B	13.58 down	8.27 down	AF078077
210512_s_at	vascular endothelial growth factor	VEGF	36.08 down	5.58 up	AF022375
213756_s_at	heat shock transcription factor 1	HSF1	8.12 up	3.47 down	AI393937
217684_at	thymidylate synthetase	TYMS	4.21 down	3.30 up	BG281679
220038_at	serum/glucocorticoid regulated kinase family. member 3	SGK3	8.24 down	9.60 down	NM_013257
**Response to wounding and wound healing**					
209277_at	Tissue factor pathway inhibitor 2	TFPI2	9.85 down	2.62 up	AL574096
203294_s_at	lectin, mannose-binding, 1	LMAN1	11.53 up	9.84 down	U09716
205767_at	epiregulin	EREG	5.13 down	3.61 down	NM_001432
209101_at	connective tissue growth factor	CTGF	595.44 down	14.43 up	M92934
**Wnt receptor activity**					
203987_at	frizzled homolog 6	FZD6	39.71 down	2.18 up	NM_003506
**Receptor complex**					
201474_s_at	integrin. alpha 3	ITGA3	5.21 down	2.36 up	NM_002204
204625_s_at	integrin. beta 3	ITGB3	2.17 up	2.47 down	BF115658
206009_at	integrin. alpha 9	ITGA9	3.18 down	3.58 down	NM_002207
211772_x_at	cholinergic receptor. nicotinic. alpha 3	CHRNA3	2.54 up	4.46 down	BC006114
204773_at	interleukin 11 receptor. alpha	IL11RA	15.25 down	2.90 down	NM_004512
**membrane**					
202637_s_at	intercellular adhesion molecule 1 (CD54)	ICAM1	28.90 down	6.61 up	AI608725
203699_s_at	deiodinase, iodothyronine, type II	DIO2	7.75 up	10.10 down	U53506
203988_s_at	fucosyltransferase 8 (alpha (1,6) fucosyltransferase)	FUT8	17.44 down	17.71 up	NM_004480
204273_at	endothelin receptor type B	EDNRB	10.11 up	2.75 down	NM_000115
205421_at	solute carrier family 22, member 3	SLC22A3	6.50 up	6.21 down	NM_021977
213856_at	CD47 molecule	CD47	12.43 down	10.98 up	BG230614
**Chemotaxis**					
205242_at	chemokine (C-X-C motif) ligand 13	CXCL13	3.03 down	7.56 up	NM_006419
209687_at	chemokine (C-X-C motif) ligand 12	CXCL12	24.98 down	2.54 up	U19495
210845_s_at	plasminogen activator, urokinase receptor	PLAUR	12.79 down	2.05 up	U08839
207850_at	chemokine (C-X-C motif) ligand 3	CXCL3	24.57 down	3.15 down	NM_002090
210163_at	chemokine (C-X-C motif) ligand 11	CXCL11	34.76 down	5.60 up	AF030514
215723_s_at	phospholipase D1, phosphatidylcholine-specific	PLD1	12.37 down	3.08 up	AJ276230
219825_at	cytochrome P450, family 26, subfamily B, polypeptide 1	CYP26B1	17.74 down	15.25 up	NM_019885
**DNA-dependent DNA replication**					
205085_at	origin recognition complex, subunit 1-like	ORC1L	10.43 down	7.25 up	NM_004153
208070_s_at	REV3-like, catalytic subunit of DNA polymerase zeta	REV3L	38,90 down	2.80 down	NM_002912
208808_s_at	high-mobility group box 2	HMGB2	5.53 down	2.41 up	BC000903
209084_s_at	RAB28, member RAS oncogene family	RAB28	23.27 down	2.09 up	BE504689
210892_s_at	general transcription factor II, i	GTF2I	3.72 down	3.64 up	BC004472
**DNA-dependent DNA replication**					
205085_at	carbonyl reductase 1	CBR1	2.39 up	2.34 down	BC002511
**G0 to G1 transition**					
205655_at	Mdm4, p53 binding protein	MDM4	3.27 down	6.07 up	NM_002393
**platelet-derived growth factor beta-receptor activity**					
205226_at	platelet-derived growth factor receptor-like	PDGFRL	5.33 up	9.50 down	NM_006207
**actin cytoskeleton organization and biogenesis**					
209209_s_at	pleckstrin homology domain containing, family C, member1	PLEKHC1	51.02 down	13.59 up	AW469573
216621_at	Rho-associated, coiled-coil containing protein kinase 1	ROCK1	8.58 down	8.52 up	AL050032
220997_s_at	diaphanous homolog 3 (Drosophila)	DIAPH3	3.72 down	10.53 up	NM_030932
208614_s_at	filamin B, beta (actin binding protein 278)	FLNB	59.49 down	2.91 down	M62994
214925_s_at	spectrin, alpha, non-erythrocytic 1 (alpha-fodrin)	SPTAN1	13.23 down	2.62 down	AK026484
215602_at	FYVE, RhoGEF and PH domain containing 2	FGD2	12.94 up	2.60 down	AK024456
**Innate immune response**					
204924_at	toll-like receptor 2	TLR2	5.94 down	3.16 up	NM_003264
206271_at	toll-like receptor 3	TLR3	55.81 down	6.59 down	NM_003265
206206_at	CD180 molecule	CD180	23.53 down	10.73 up	NM_005582
210166_at	toll-like receptor 5	TLR5	27.49 down	2.03 up	AF051151
215388_s_at	complement factor H	CFH	561.66 down	2.36 down	X56210
206157_at	pentraxin-related gene, rapidly induced by IL-1 beta	PTX3	5.17 down	12.45 up	NM_002852
206693_at	interleukin 7	IL7	3.06 down	11.97 up	NM_000880
206727_at	complement component 9	C9	6.43 up	9.24 down	K02766

SSc, progressive systemic sclerosis

Noteworthy is that most of these genes showed a significant change at transcription level after iloprost infusion.

Among genes related to apoptosis, for instance, anti-apoptotic genes such as RAS p21 protein activator 1 (RASA1), protein-kinases, AMP-activated alpha1 (PRKAA1) and BCL2 interacting protein 3 (BNIP3) were down-regulated in sclerodermic patients (F.C. -8.72, -6.49 and -69.05 respectively) but up-regulated after treatment (F.C. + 4.29, + 6.61, + 11.78).

Genes involved in the cellular response to stress had a similar behaviour; CD59, a complement regulatory protein, was strongly down-regulated in SSc patiens (F.C. -18.77) and up-regulated by the treatment (F.C. + 2.72). Vascular endothelial growth factor (VEGF) a well-known mitogen for vascular endothelial cells and a fundamental molecule for the EPCs recruitment from bone marrow, was greatly repressed in SSc patients (FC -36.08) but highly induced (F.C. + 5.58) after iloprost treatment.

The high increase of heat shock transcription factor 1 (HSF1) (F.C. + 8.12) was followed by a marked reduction (F.C. -3.47) after iloprost treatment.

Another cluster of modulated genes was represented by genes involved in the process of wounding and wound healing. Tissue factor pathway inhibitor-2 (TFPI2) is regulated by vascular endothelial growth factor and indeed its expression profile varied similarly to VEGF (F.C. -9.85 before and F.C. + 2.62 after iloprost). Indeed connective tissue growth factor (CTGF) showed the strongest down-regulation in SSc patients (F.C. -595.44) which was followed by a marked up-regulation (F.C. + 14.43) after treatment.

Iloprost also influenced the adhesion properties of CECs since several integrin genes were modulated in SSc patients after treatment. Expression level of intercellular adhesion molecule 1 (ICAM1) varied from a down-regulation of -28.91 F.C. to an up-regulation of + 6.61 F.C. The transcription level of endothelin receptor type B (EDNRB) gene varied from F.C. + 10.11 to F.C. -2.75.

The functional class named chemotaxis included genes encoding for chemokines, a group of molecules able to attract leukocytes and regulate angiogenesis, vascular proliferation and fibrosis. Several genes encoding for chemokines (CXCL13, CXCL12; CXCL3, CXCL11) had a significant change at the transcription level after iloprost infusion.

The CECs transcriptome modulated by iloprost treatment was also enriched in transcripts involved in the innate immune response regulation. This functional class included several toll like receptors (TLR2, 3 and 5) in particular TLR3 and TLR5 expression underwent extensive variation in SSc patients after iloprost infusion (F.C. -55.82 and -27.49 before treatment to F.C. -6.59 and + 2.03 after treatment).

A very strong reduction in expression (F.C. -561.66) of the gene encoding for complement factor H (CFH) was observed in CECs during SSc, however such reduction was less pronounced (F.C. -2.36) after iloprost infusion.

The same analysis was performed on CECs isolated from SSc patients without digital ulcers. Therefore we focused our attention on the genes significantly modulated in SSc patients, whose expression was also influenced by iloprost treatment.

Using these criteria we identified 3,990 genes, which were stratified over a large number of different functional classes of genes. The results are presented in compiled form in Table 5, bold characters indicate genes also present in SSc with digital ulcers. A large number of such transcripts were ascribed to the same functional classes analyzed for SSc with digitals ulcers. We found that genes belonging to these GO categories were therefore modulated in both disease subsets (with or without digital ulcers).

**Table 5:** Functional classification of genes modulated by iloprost in SSc patients without digital ulcers

Probe Set ID	Gene Title	Gene symbol	FC SSc/healthy	FC SSc post-treatment/SSc pre-treatment	Representative Public ID
**Positive regulation of anti-apoptosis**					
201849_at	BCL2/adenovirus E1B 19 kDa interacting protein 3	**BNIP3**	19.65 down	5.93 up	NM_004052
210621_s_at	RAS p21 protein activator (GTPase activating protein) 1	**RASA1**	5.35 down	2.02 up	M23612
214917_at	protein kinase, AMP-activated, alpha 1 catalytic subunit	**PRKAA1**	2.88 down	3.50 up	AK024252
**Response to stress**					
202906_s_at	nibrin	**NBN**	3.83 down	4.33 up	AF049895
206040_s_at	mitogen-activated protein kinase 11	**MAPK11**	3.53 up	2.15 up	NM_002751
209305_s_at	growth arrest and DNA-damage-inducible, beta	**GADD45B**	11.23 down	4.25 up	AF078077
210512_s_at	vascular endothelial growth factor	**VEGF**	7.03 down	2.38 up	AF022375
217684_at	thymidylate synthetase	**TYMS**	2.76 down	5.84 up	BG281679
**Response to wounding and wound healing**					
209101_at	connective tissue growth factor	**CTGF**	1912.1 down	11.18 up	M92934
209277_at	Tissue factor pathway inhibitor 2	**TFPI2**	3.36 down	8.90 down	AL574096
**Wnt receptor activity**					
203987_at	frizzled homolog 6	**FZD6**	19.85 down	3.25 up	NM_003506
**Receptor complex**					
201474_s_at	integrin, alpha 3	**ITGA3**	3.37 down	2.04 up	NM_002204
206009_at	integrin, alpha 9	**ITGA9**	2.05 down	2.02 up	NM_002207
211772_x_at	cholinergic receptor, nicotinic, alpha 3	**CHRNA3**	2.10 up	12.73 down	BC006114
204773_at	interleukin 11 receptor, alpha	**IL11RA**	10.76 down	6.66 up	NM_004512
**Membrane**					
202638_s_at	intercellular adhesion molecule 1 (CD54)	**ICAM1**	21.89 down	2.00 up	NM_000201
204273_at	endothelin receptor type B	**EDNRB**	12.18 up	9.07 up	NM_000115
205421_at	solute carrier family 22, member 3	**SLC22A3**	14.37 up	8.31 down	NM_021977
213857_s_at	CD47 molecule	**CD47**	6.17 down	3.51 up	BG230614
**Chemotaxis**					
207850_at	chemokine (C-X-C motif) ligand 3	**CXCL3**	35.50 down	2.32 up	NM_002090
211122_s_at	chemokine (C-X-C motif) ligand 11	**CXCL11**	2.98 down	2.32 down	AF002985
215723_s_at	phospholipase D1, phosphatidylcholine-specific	**PLD1**	7.72 down	2.07 up	AJ276230
219825_at	cytochrome P450, family 26, subfamily B, polypeptide 1	**CYP26B1**	25.09 down	3.98 down	NM_019885
203218_at	mitogen-activated protein kinase 9	MAPK9	9.53 down	7.02 up	W37431
**DNA-dependent DNA replication**					
208070_s_at	REV3-like, catalytic subunit of DNA polymerase zeta	**REV3L**	21.36 down	8.90 up	NM_002912
208808_s_at	high-mobility group box 2	**HMGB2**	2.78 down	3.18 up	BC000903
209084_s_at	RAB28, member RAS oncogene family	**RAB28**	7.25 down	3.79 up	BE504689
213336_at	General transcription factor II, i	**GTF2I**	4.51 down	4.52 up	AI826454
**Prostaglandin-E2 9-reductase activity**					
50221_at	transcription factor EB	TFEB	6.09 down	11.09 up	AI524138
**G0 to G1 transition**					
210386_s_at	metaxin 1	MTX1	6.35 down	4.85 up	BC001906
**platelet-derived growth factor beta-receptor activity**					
205226_at	platelet-derived growth factor receptor-like	**PDGFRL**	5.04 up	2.72 up	NM_006207
**actin cytoskeleton organization and biogenesis**					
208614_s_at	filamin B, beta (actin binding protein 278)	**FLNB**	92.27 down	2.75 up	M62994
214925_s_at	spectrin, alpha, non-erythrocytic 1 (alpha-fodrin)	**SPTAN1**	17.80 down	8.95 up	AK026484
215602_at	FYVE, RhoGEF and PH domain containing 2	**FGD2**	15.22 down	5.67 up	AK024456
**Innate immune response**					
206271_at	toll-like receptor 3	**TLR3**	8.66 down	10.24 down	NM_003265
210166_at	toll-like receptor 5	**TLR5**	7.83 down	2.95 up	AF051151
215388_s_at	complement factor H	**CFH**	295.64 down	3.15 down	X56210
206693_at	interleukin 7	**IL7**	10.94 down	2.96 up	NM_000880
203854_at	complement factor I	CFI	578.23 down	16.50 up	NM_000204
206727_at	complement component 9	**C9**	11.81 down	10.01 up	K02766

SSc, progressive systemic sclerosis

Noteworthy was that most of the selected genes had a similar response to iloprost infusion when compared to the other disease subset. The results further confirm that iloprost treatment exerts a strong effect on the transcriptional profiles of CECs obtained from SSc patients.

Finally, we compared the gene expression profiles of CECs from the two subsets of SSc patients and found that 2,303 genes were significantly modulated in SSc with digital ulcers as compared to SSc without digital ulcers. The Gene Ontology analysis of these transcripts revealed a functional enrichment (*P *< 0.02) in several gene categories including immune response, response to wounding and inflammatory response (Table 6).

**Table 6:** Functional enrichment in gene categories in patients with skin ulcers compared to those without ulcers

Biological process	Number of probe sets	*P*-value
Immune response	210	0.0000000009
Defense response	221	0.0000000145
Response to wounding	109	0.0000012730
Signal transducer activity	472	0.0000206037
Response to stress	236	0.0000215677
Regulation of cell proliferation	89	0.0000403309
Positive regulation of nitric oxide biosynthesis	8	0.0001150065
Positive regulation of biosynthesis	16	0.0001813324
Receptor activity	290	0.0003570920
Cell activation	36	0.0005153290
Cell proliferation	137	0.001020579
Positive regulation of innate immune response	3	0.001302911
Regulation of immune response	27	0.001521656
Inflammatory response	47	0.002011958
Positive regulation of immune response	20	0.002900865
Cell adhesion	136	0.006908177
Cytokine activity	47	0.017808983

Interestingly, iloprost treatment modulated 59.5% of these transcripts (1,370/2,303).

These data show that there is a significant difference in the trascriptional profiles of CECs isolated from SSc patients with or without digital ulcers. The results therefore indicate that CECs are quite heterogeneous within the same disease and that these differences may be associated to the presence of a particular clinical subset.

### Real Time RT-PCR validation of gene array results

**Figure 2:**
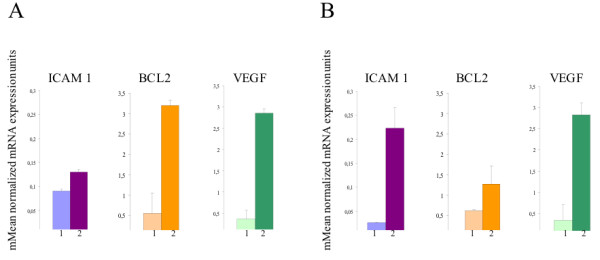
**Validation of gene array results by Real Time RT-PCR**. Genes selected for validation by Real Time RT-PCR in CECs before (bar 1) and after (bar 2) iloprost infusion in SSc patients without **(panel A) **and with **(panel B) **digital ulcers. ICAM-1, Bcl2 and VEGF transcripts were increased several times after iloprost infusion in both groups of patients. The increase was statistically significant (*P *< 0.05) in all cases. The experiments were carried out in triplicates.

We validated the results obtained with the gene array by Real Time RT-PCR using the same endothelial total RNA extract that was used for the gene array analysis. The Real Time RT-PCR results were concordant with the array results in three of three genes tested in the two subsets studied, in terms of significant differences in gene expression between CECs derived from patients affected by SSc with and without skin ulcers before and after iloprost infusion. The genes subjected to validation included those encoding VEGF, ICAM-1 and BCL-2 (Figure 2). GAPDH was selected as endogenous standard, and we saw no significant changes in the Q-PCR results when the data were normalized using beta-actin, another constitutively transcribed gene.

## Discussion

We have detected and quantified CECs and EPCs in the peripheral blood of 50 SSc patients using a four-color flow-cytometry approach. The gating strategy and the presence of a dump channel allows the detection of both CECs and EPCs with high precision and a high intra-assay reproducibility. Moreover, we have followed the EULAR recommendations on endothelial precursor cells quantification [42]. Most of the reports on CECs and EPCs enumeration have used a three-color flow-cytometry [6,43] and different markers from those recommended by EULAR explaining the controversial results obtained by different groups [6,44]. We needed a precise enumeration of CECs and EPCs also because we had to use them for the gene array study.

In our cohort of SSc patients, the number of both CECs and EPCs was higher than in healthy donors as already reported [6]. The increased EPC levels in SSc support their mobilisation from bone marrow in the attempt of revascularization in response to vascular ischemia. Moreover the counts of CECs correlated with the clinical stage of the disease, since a higher number was detectable in patients with a more severe vascular damage (presence of digital ulcers). Patients with digital vascular lesions did not show a significant increased number of EPCs in accordance with previous data [45] and suggesting an increased homing at this stage.

We observed that iloprost infusion significantly increased the number of both cell types in all the patients treated. To our knowledge, the finding of increased levels of CECs and EPCs in patients with SSc after iloprost treatment has not been previously reported and may be of difficult interpretation since one would expect a reduction of these cells to the levels similar to those seen in healthy controls. A possible explanation for these findings is that iloprost infusion may be responsible for the *in vivo *recruitment of EPCs from bone marrow and for their homing into sites of angiogenesis and/or vascular damage, thus contributing to neovascularization and/or wound-healing processes. Moreover, the drug may favour the migration and proliferation of mature endothelial cells surrounding the sites of vascular damage thus leading to an increase shedding of damaged cells. However, the increase of EPCs is not confined to iloprost therapy since a statistically significant increase in EPCs has also been observed during atorvastatin treatment in patients with SSc [43].

In SSc patients, CECs were not only increased in their number but also revealed a completely different transcriptional profile when compared to that of CECs obtained from healthy donors. We decided to focus our attention on the different expression of genes strictly related to vasculogenesis, reparative processes, cell migration and homing, since a deficient vascular repair mechanism and defective vasculogenesis are the main contributors to vasculopathy in Ssc.

We found a significant decrease in expression of genes encoding for molecules involved in the negative regulation of apoptosis (RASA1, PRKAA1 and BNIP3) suggesting that the cells are prone to apoptosis. Apoptosis of endothelial cells is considered the primary pathogenic event in SSc and the downmodulation of genes encoding for antiapoptotic molecules helps in understanding the molecular basis of this event. Moreover, the downregulation of SGK3, also called cytokine-independent survival kinase (CISK), a survival kinase involved in cellular response to stress protecting cells from apoptosis [46], further indicates an impaired regulation of cellular survival pathways.

In SSc patients CECs showed also a decreased transcription of TFPI2, EREG and CTGF, molecules crucially involved in tissue-specific proliferation/differentiation homeostasis and effective reparative activity, indicating that these cells are also compromised in their wound healing capacity. CTGF, a growth factor produced as part of a growth factor cascade during vascular injury responses, can also modulate the activity of angiogenic molecules such as VEGF [47,48] and similarly, VEGF is a potent inducer of CTGF mRNA [49,50]. Therefore the simultaneous under-expression of both genes is not surprising.

Another function severely compromised in SSc is the adherence ability of CECs, demonstrated by the reduced transcription levels of several adhesion molecules such as ICAM1 and integrins. This aspect together with the downregulation of different chemokines may suggest a reduced cell migration and therefore a reduced andothelial cell homing. In particular, the reduced expression of CXCL12 (SDF1) in patients with digital ulcers is in accordance with previous findings on the reduced expression of this chemokine in late phase of the disease [51].

Endothelin-1 and its receptors A and B play a pivotal role both in vasoconstriction and in fibrosis. Receptors A are expressed on smooth muscle cells and mediate vasoconstriction, whereas receptors B are expressed on endothelial cells and mediates primarily vasodilatation through nitric oxide production. The upregulation of endothelin-1 receptor B gene expression by CECs suggests the attempt of endothelial cells to privilege the vasoactive effect and to increase tissue blood flow.

VEGF, a potent angiogenic mitogen playing a crucial role in angiogenesis under various pathophysiological conditions, was strongly down-regulated probably in relation to a reduced proangiogenic activity of CECs in SSc. Interestingly VEGF can mobilize EPCs from bone marrow and accelerate endothelial damage repair [52,53]. Therefore the increase in VEGF gene expression after iloprost therapy may explain the EPCs increase in an attempt to repair the vascular damage. The increase of the VEGF gene expression after iloprost stimulation has already been described in lung fibroblasts [24]. Moreover iloprost has been reported to induce VEGF production from intestinal epithelial cells [54], from monocytes [55] and to modulate VEGF secretion from platelets isolated from SSc patients [56]. It is well known that VEGF has been found elevated in the sera of patients with SSc. However, we must consider first of all that the gene array analysis has been carried out in CECs, which can exhibit a different transcriptional profile from that expected and seen in the same cells in different conditions, that is, cells isolated from skin and coltured [57] or in a whole tissue [58]. Secondly, VEGF is produced from different cell types as mentioned above and this may account at least in part for the increased levels of circulant VEGF in patients with SSc. As for VEGF gene expression, iloprost infusion showed a strong effect on the transcriptional profiles of CECs isolated from patients with SSc both in the presence or absence of digital ulcers, since it induced a marked modulation of the differently expressed genes.

When we considered the genes over- and under-expressed in SSc patients versus healthy donors we found that the treatment induced an opposite behaviour of most of these transcripts. Thus several molecular functions repressed in CECs of scleroderma patients, were restored after iloprost infusion as shown by the over-expression of antiapoptotic genes (RASA1, PRKAA1 and BNIP3) and of the transcripts encoding for adhesion molecules (ICAM1, ITGA3, ITGA9), chemokines and wound healing process (EREG, CTGF). Also, the decrease in heat shock transcription factor 1 expression after iloprost treatment is in accordance with the protective effects of the drug on endothelial cells. The gene array data concerning the drug modulation of genes involved in the control of apoptosis, in cell adhesion and in vasculogenesis were validated by quantitative RT-PCR of selected genes (BCL2, ICAM1, VEGF) in SSc patients with and without digital ulcers.

## Conclusions

In this study we analyzed gene expression profiles of CECs and EPCs obtained from healthy subjects and from SSc patients with and without digital ulcers before and after iloprost treatment using a gene array approach. Based on gene ontology analysis we found that CECs from patients show down-modulation of genes involved in the control of apoptosis, in cell migration and adhesion and in angiogenesis. Several impaired cellular function were reversed by iloprost treatment and the gene array modulation was validated by quantitative RT-PCR of selected genes. The different expression profile of CECs in SSc patients compared to normal subjects account for endothelial cell apoptosis and for the impaired angiogenesis in the disease. Moreover, our data give a novel insight into the vascular repairing effects of iloprost treatment.

## Abbreviations

CECS: circulating endothelial cells; CTGF: connective tissue growth factor; EPCS: endothelial progenitor cells; FACS: fruorescence activated cell sorting; SSC: progressive systemic sclerosis; VEGF: vascular endothelial growth factor;

## Competing interests

The authors declare that they have no competing interests.

## Authors' contributions

TE enrolled the patients and controls, made the FACS analysis of endothelial cells and provided their isolation from peripheral blood and to endothelial cells' RNA extraction. She also provided the statistical analysis. MD and PA performed the gene array analysis. RA helped in the flow-cytometric analysis of endothelial cells. LC and PA were responsible for the project and wrote the manuscript with input from CR. BR and VMT performed the Real Time RT-PCR. All the authors have read and approved the final manuscript.

## Supplementary Material

Additional file 1 **Gene expression profile in healthy subjects**. Raw intensity signal values obtained from the sample of healthy subjects. Click here for file

Additional file 2 **Gene expression profile in SSc patients with ulcers**. Raw intensity signal values present in the sample obtained from SSc patients with ulcers. Click here for file

Additional file 3 **Modulated genes in circulating endothelial cells of patients with ulcers compared to normal controls**. Fold change values obtained from the comparison between the expression levels of genes in circulating endothelial cells of patients with skin ulcers and those of normal controls. Click here for file

Additional file 4 **Gene expression profile in SSc patients without ulcers**. Raw intensity signal values obtained from the sample of SSc patients without skin ulcers. Click here for file

Additional file 5 **Genes modulated in SSc patients without ulcers compared to healthy donors**. Fold change values obtained from the comparison between the expression levels of genes in circulating endothelial cells of patients without ulcers and those of healthy donors. Click here for file

Additional file 6 **Gene expression profile in SSc patients with ulcers after treatment**. Raw intensity signal values obtained from the sample of SSc patients with ulcers after treatment with Iloprost. Click here for file

Additional file 7 **Genes modulated by Iloprost treatment in patients with skin ulcers**. Fold change values of modulated genes obtained from the comparison between the expression levels of genes in circulating endothelial cells of patients with skin ulcers after Iloprost treatment and those of the same patients before Iloprost treatment. Click here for file

Additional file 8 **Gene expression profile in SSc patients without ulcers after Iloprost treatment**. Raw intensity signal values of the sample obtained from SSc patients without skin ulcers after treatment. Click here for file

Additional file 9 **Genes modulated by Iloprost treatment in patients without skin ulcers**. Fold change values obtained from the comparison between the expression levels of genes in circulating endothelial cells of patients without skin ulcers after Iloprost treatment and those of the same patients before Iloprost treatment. Click here for file
